# Supplementary material for: On the Primary Water Radicals’ Production in the Presence of Gold Nanoparticles: Electron Pulse Radiolysis Study
Source: Nanomaterials (Basel). 2020 Dec 10;10(12):2478. doi: 10.3390/nano10122478 (PMC7763946; doi:10.3390/nano10122478)
Supplement: Supplementary file 1 [file nanomaterials-10-02478-s001.pdf]

Supplementary Information

On the Primary Water Radicals' Production in the Presence of Gold Nanoparticles: Electron Pulse Radiolysis Study

Viacheslav Shcherbakov, Sergey A. Denisov * and Mehran Mostafavi *

Institute de Chimie Physique (ICP), CNRS/Université Paris-Saclay, Bât. 349, 91405 Orsay, France; viacheslav.shcherbakov@universite-paris-saclay.fr

* Correspondence: sergey.denisov@universite-paris-saclay.fr (S.A.D.); mehran.mostafavi@universite-paris-saclay.fr (M.M.)

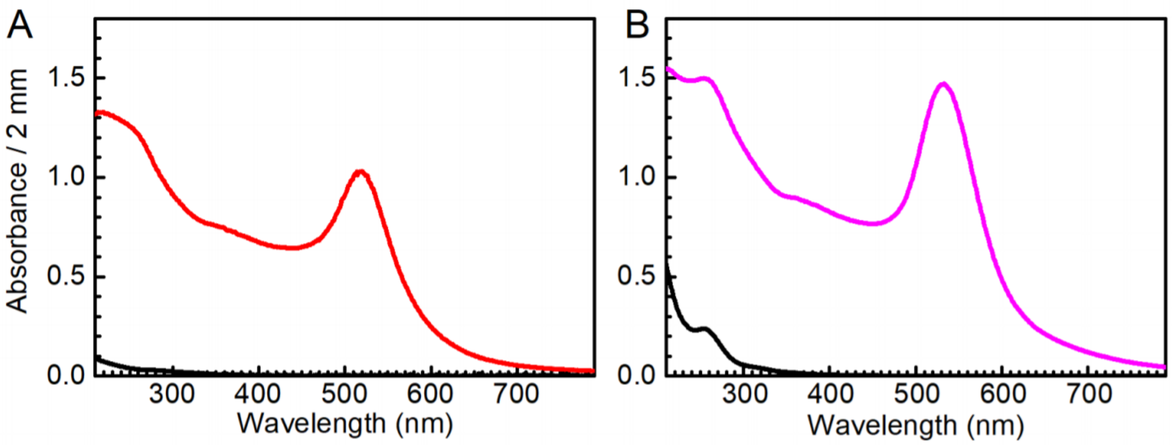

Figure S1. Absorption spectra of AuNPs suspensions containing 3 mM of gold atoms prepared by borohydride reduction method (red), and its supernatant (black) (**A**); Turkevich method (pink), and its supernatant (black) (**B**), the peak at 260 nm is due to the presence of acetonedicarboxylic acid, a product of citrate oxidation. AuNPs' suspensions were diluted with deionized water two times before measurement.

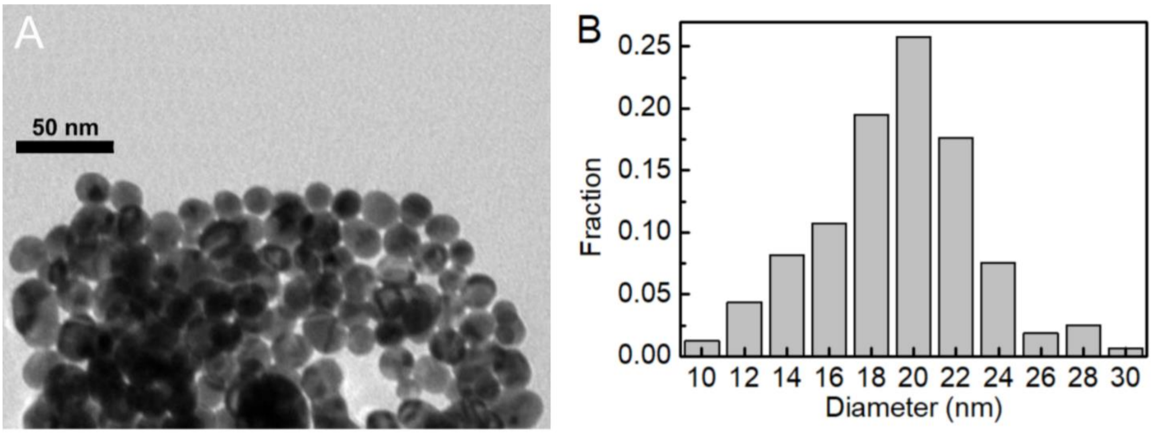

Figure S2. Characterization of AuNPs prepared by borohydride reduction method. TEM image (**A**) and size distribution (**B**).

Silver Nanoparticles Synthesis

Silver nanoparticles (AgNPs) were synthesized by reducing silver ions by sodium borohydride and sodium citrate (Turkevich method). In both cases, the concentration of silver atoms was 2 mM. The synthesis using borohydride included the following steps: 12 mL of 10 mM AgClO4 were mixed with 1.2 mL of 100 mM sodium citrate (used as a stabilizer) and 44.4 mL of deionized water at room temperature. Then 2.4 mL of 100 mM NaBH4 was adding under stirring by magnet stir bar with high speed. Stirring was continued 10 min after borohydride adding. The natural pH of the solution was 9. Preparation of AgNPs by Turkevich method included the following steps: 14 mL of 10 mM AgClO4 were diluted in 53.2 ml of deionized water. The solution was heated up to boiling. Then 2.8 mL of 100 mM sodium citrate was added under stirring. After that, heating was turned off, and stirring was continued for 10 min. The natural pH of the solution was 7.5. All chemicals were purchased from Sigma-Aldrich (St. Quentin Fallavier, France).

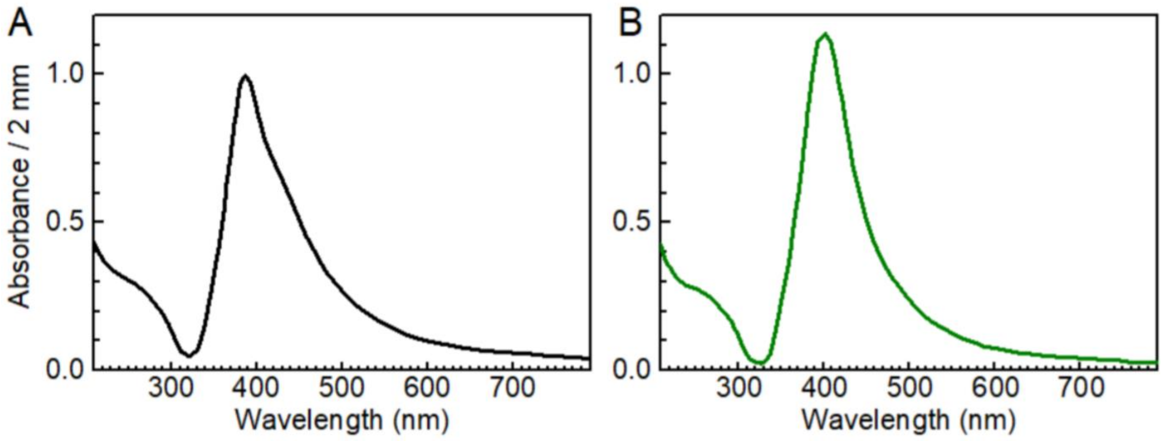

Figure S3. Absorption spectra of AgNPs prepared by borohydride reduction method (**A**), and Turkevich method (**B**). AgNPs' suspensions were diluted with deionized water 4 times before measurements.

Pulse-Radiolysis of AgNPs Suspension

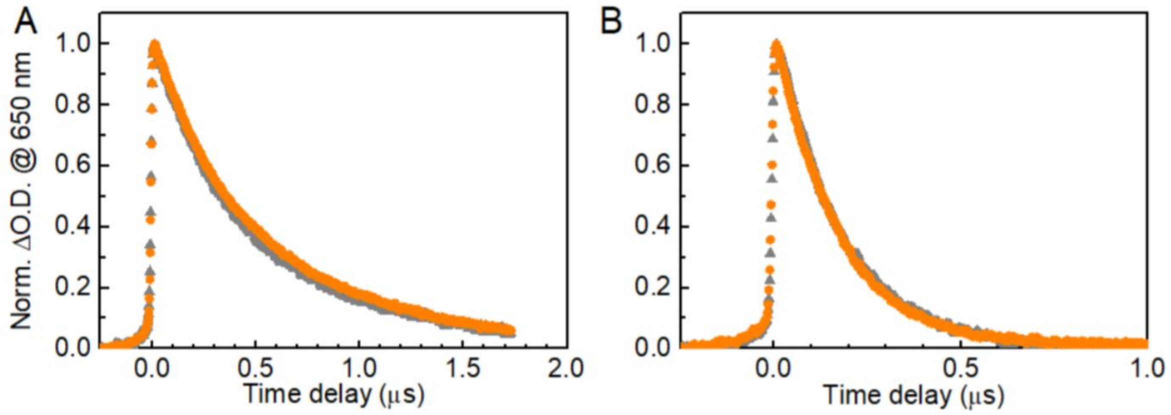

Figure S4. Kinetics of hydrated electrons within microsecond time range after a 5 ps electron pulse in AgNPs suspensions containing 2 mM of silver atoms (orange dots) and their supernatants (grey triangles). (**A**) AgNPs were prepared by the borohydride reduction method. (**B**) AgNPs were prepared by the Turkevich method.

Additional Results with AuNPs

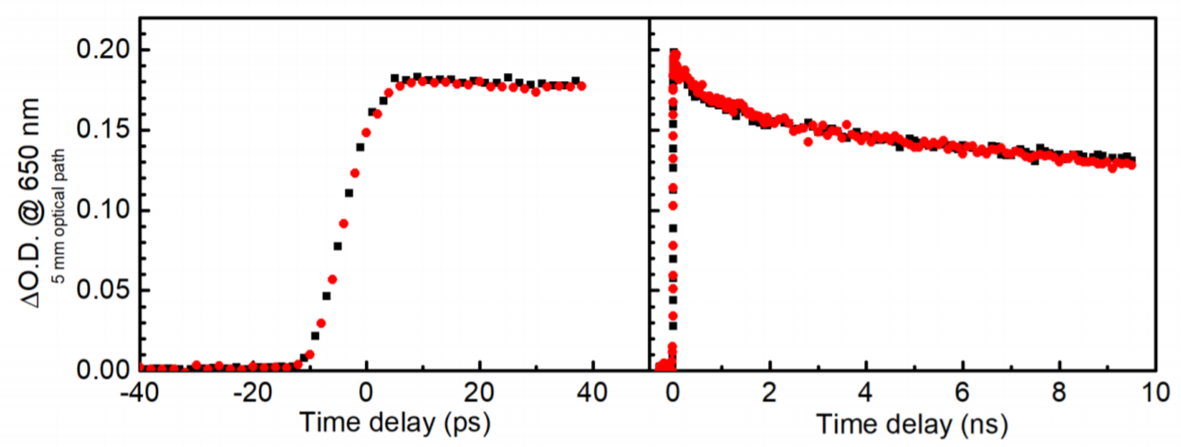

Figure S5. Kinetics of hydrated electrons within picosecond and nanosecond time ranges after a 5 ps electron pulse in water (black squares) and AuNPs suspension containing 2 mM of gold atoms prepared by borohydride reduction method (λ_{max} = 518 nm) (red dots).

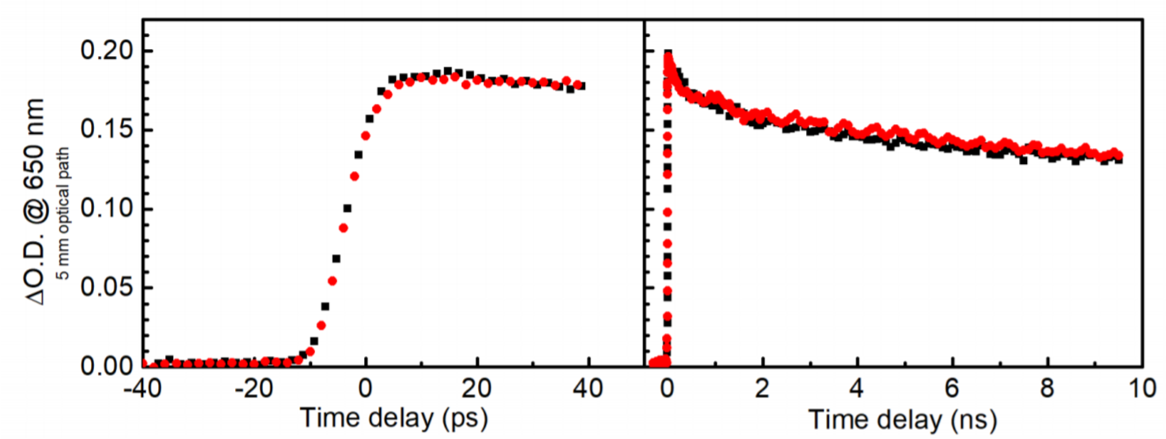

Figure S6. Kinetics of hydrated electrons within picosecond and nanosecond time ranges after a 5 ps electron pulse in water (black squares) and AuNPs suspension containing 2 mM of gold atoms prepared by borohydride reduction method with 3 mM of sodium citrate used as a surfactant (λ_{max} = 517 nm) (red dots). No effect of additional citrate (3 mM) was observed.

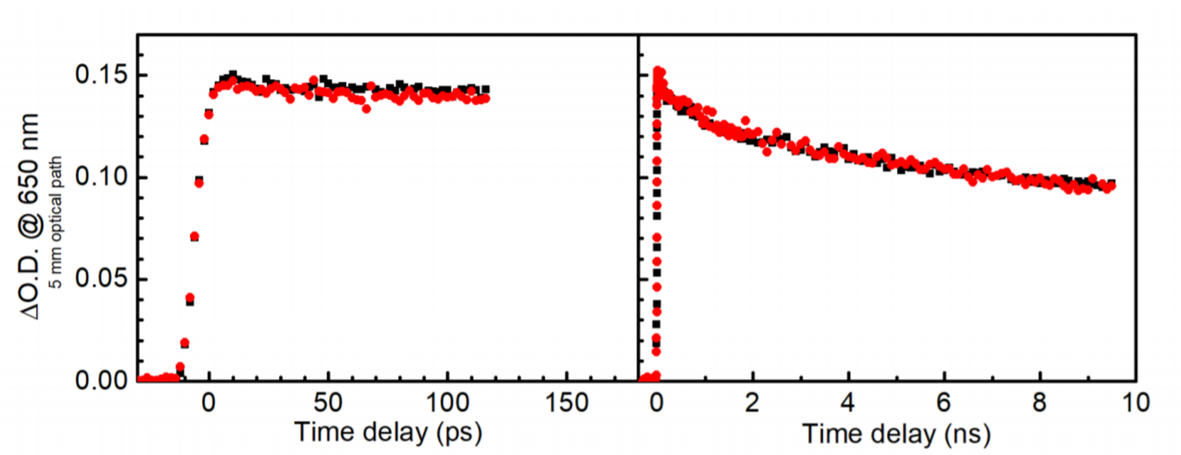

Figure S7. Kinetics of hydrated electrons within picosecond and nanosecond time ranges after a 5 ps electron pulse in water (black squares) and AuNPs suspension containing 2 mM of gold atoms prepared by the Turkevich method (Au:citrate ratio is 1:1.5, λ_{max} = 533 nm) (red dots).

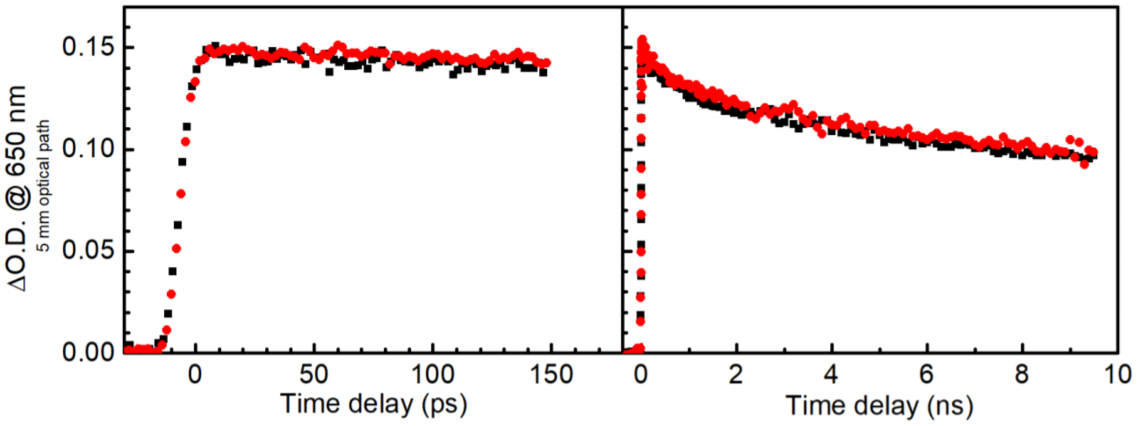

Figure S8. Kinetics of hydrated electrons within picosecond and nanosecond time ranges after a 5 ps electron pulse in water (black squares) and AuNPs suspension containing 1 mM of gold atoms prepared by the Turkevich method (Au:citrate ratio is 1:1.5, λ_{max} = 533 nm) (red dots).

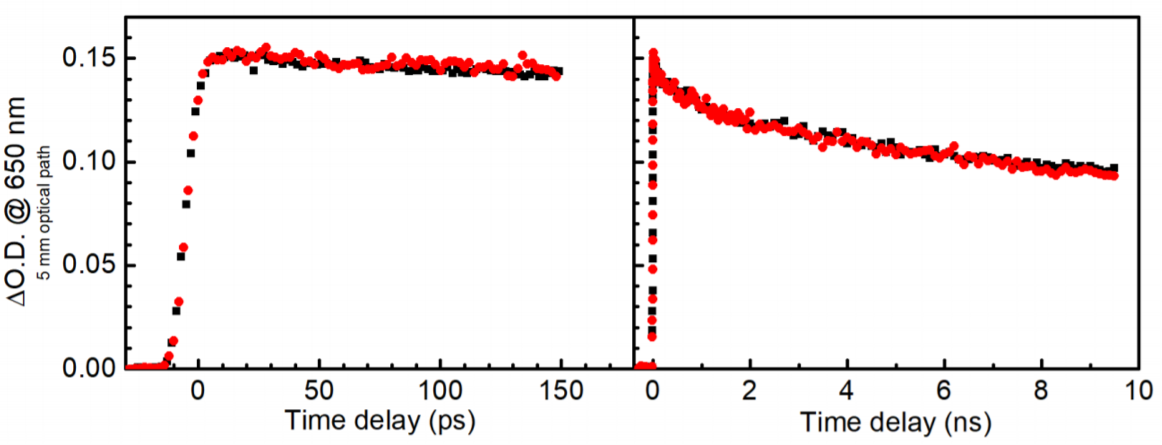

Figure S9. Kinetics of hydrated electrons within picosecond and nanosecond time ranges after a 5 ps electron pulse in water (black squares) and AuNPs suspension containing 2 mM of gold atoms prepared by the Turkevich method (Au:citrate ratio is 1:4, λ_{max} = 520 nm) (red dots).

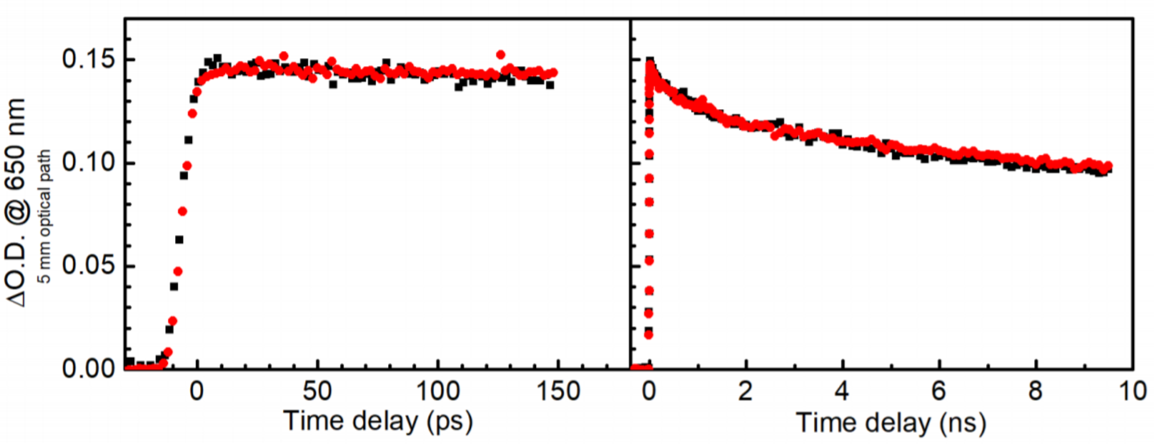

Figure S10. Kinetics of hydrated electrons within picosecond and nanosecond time ranges after a 5 ps electron pulse in water (black squares) and AuNPs suspension containing 1 mM of gold atoms prepared by the Turkevich method (Au:citrate ratio is 1:4, λ_{max} = 520 nm) (red dots).
